# A cluster randomized trial to assess the effect of clinical pathways for patients with stroke: results of the clinical pathways for effective and appropriate care study

**DOI:** 10.1186/1741-7015-10-71

**Published:** 2012-07-10

**Authors:** Massimiliano Panella, Sara Marchisio, Romeo Brambilla, Kris Vanhaecht, Francesco Di Stanislao

**Affiliations:** 1 Department of Clinical and Experimental Medicine, University of Eastern Piedmont 'A. Avogadro', Novara, Italy; 2 Unit for Quality Improvement, Health Authority ASL VC, Vercelli, Italy; 3 Unit of Epidemiology, School of Public Health, Grugliasco, Turin, Italy; 4 Center for Health Services and Nursing Research, School of Public Health, Catholic University Leuven, Leuven, Belgium; 5 The European Pathway Association, Leuven, Belgium

## Abstract

**Background:**

Clinical pathways (CPs) are used to improve the outcomes of acute stroke, but their use in stroke care is questionable, because the evidence on their effectiveness is still inconclusive. The objective of this study was to evaluate whether CPs improve the outcomes and the quality of care provided to patients after acute ischemic stroke.

**Methods:**

This was a multicentre cluster-randomized trial, in which 14 hospitals were randomized to the CP arm or to the non intervention/usual care (UC) arm. Healthcare workers in the CP arm received 3 days of training in quality improvement of CPs and in use of a standardized package including information on evidence-based key interventions and indicators. Healthcare workers in the usual-care arm followed their standard procedures. The teams in the CP arm developed their CPs over a 6-month period. The primary end point was mortality. Secondary end points were: use of diagnostic and therapeutic procedures, implementation of organized care, length of stay, re-admission and institutionalization rates after discharge, dependency levels, and complication rates.

**Results:**

Compared with the patients in the UC arm, the patients in the CP arm had a significantly lower risk of mortality at 7 days (OR = 0.10; 95% CI 0.01 to 0.95) and significantly lower rates of adverse functional outcomes, expressed as the odds of not returning to pre-stroke functioning in their daily life (OR = 0.42; 95 CI 0.18 to 0.98). There was no significant effect on 30-day mortality. Compared with the UC arm, the hospital diagnostic and therapeutic procedures were performed more appropriately in the CP arm, and the evidence-based key interventions and organized care were more applied in the CP arm.

**Conclusions:**

CPs can significantly improve the outcomes of patients with ischemic patients with stroke, indicating better application of evidence-based key interventions and of diagnostic and therapeutic procedures. This study tested a new hypothesis and provided evidence on how CPs can work.

**Trial registration:**

ClinicalTrials.gov ID: [NCT00673491].

## Background

Stroke represents one of the major public-health issues worldwide [1-4]. The American Stroke Association has reported that obstacles in translating scientific advances into clinical practice are often related to fragmentation caused by inadequate integration of facilities and professionals that should closely collaborate [5]. This potentially contributes to the high morbidity, mortality, and economic cost of stroke [5-7]. Other studies have suggested that establishing well-organized, multidisciplinary care can help improve the quality of the service delivered and reduce the mortality rates associated with stroke [8,9]. According to the Helsingborg Declaration of 2006 on European Stroke Strategies, all patients should have access to a continuum of care, from stroke units in the acute phase to appropriate rehabilitation and secondary prevention measures [10]. Consequently, to improve the outcome, research and development priorities were identified: the optimization of the use of multidisciplinary teams, the development of better ways to deliver education to professionals and the public, the implementation of evidence-based care, and the evaluation of different models of stroke services [10].

The Australian National Stroke Foundation suggests that all patients with stroke who are admitted to the hospital should be managed using a clinical pathway (CP) [11]. This recommendation was made using a body of evidence from the Cochrane Collaboration but care should be taken in its application [11-13]. Despite continuing interest in implementing CPs, the evidence base for their effectiveness remains inconclusive [14]. Therefore, the purpose of the Clinical Pathways for Effective and Appropriate Care (CPEAC) Study (ClinicalTrials.gov number: NCT00673491) was to determine whether CPs can improve quality of care. The main objective was to determine whether CPs are more effective than usual care in treating patients with stroke, and whether CPs reduce both patient mortality and improve patient outcomes. The secondary objectives were to determine whether CPs increase the appropriateness of the care provided and to determine whether CPs help in implementing organized care in stroke-care facilities.

## Methods

### Ethics approval and funding

The project received ethical clearance as a prerequisite of approval for funding from the Italian Ministry of Health. The CPEAC study was approved by the ethics committe of Ancona, Marche Region, Italy, and the research was carried out in compliance with the Declaration of Helsinki. The managers in each unit consented to their clinic taking part in the trial. Patient consent to be randomized to the intervention or control arms was not obtained, because the study design required randomization to occur at the unit level. However, all individual patients gave consent to participate in the study and had the opportunity to withdraw from the study at any time. All patient data were managed in accordance with the Italian Data Protection Act [15]. The CPEAC Study was promoted and funded by the Italian Ministry of Health (Special Programs art. 12 bis D.lgs 229/99) and Marche Region [15].

### Design overview

The CPEAC Study was designed as a multicenter cluster randomized controlled trial (cRCT), in which patients with stroke were randomized either to the experimental (CP) group or to the non-intervention (usual care; UC) group [15]. The study was designed in accordance with the CONSORT statement for cRCTs [16]. It was carried out during the period July 2005 to May 2007, and involved a sample of Italian hospitals. A cluster design was used because of the ethical and logistical issues associated with the implementation of CPs, which involves a series of complex actions at the institutional level [17-23]. A pilot study to define baseline levels of performance has been described previously [24].

### Setting

**Figure 1:**
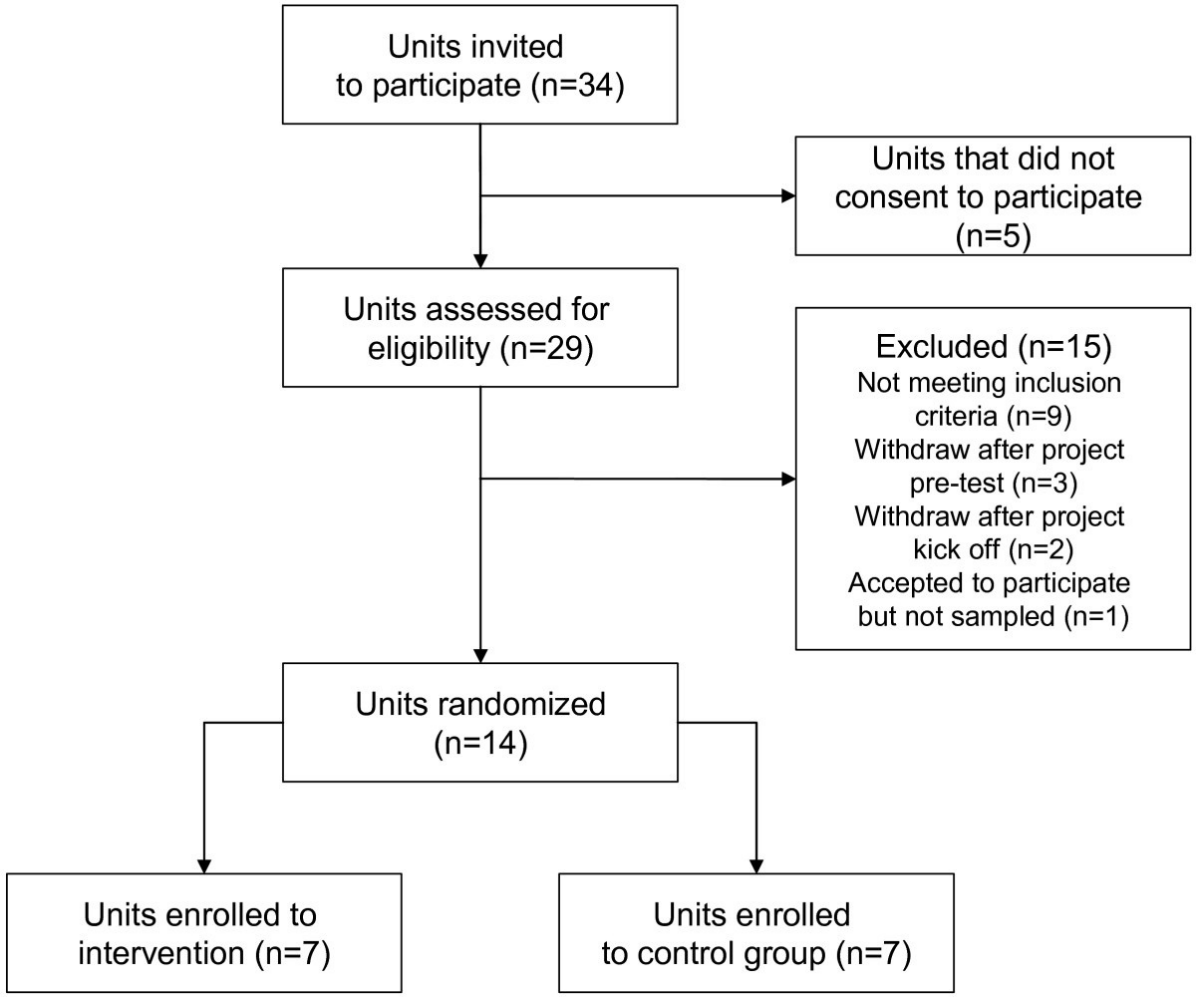
**Overview of randomization**.

Thirty-four units based in nine Italian regions were invited to participate in the study (Figure 1); of these, twenty-nine units expressed interest in implementing stroke CPs, and were assessed for eligibility [15]. The selection of the units and the final randomization was based on the comparability of their location, patient population and volume, facilities, and teaching status. To participate in the study, the administrators of the units had to allow the institution to be allocated to either of the two research groups (CP or current practice) for a period of 1 year, and had to agree not to implement a CP for the acute care and/or rehabilitation of stroke if their institution was assigned to the UC group. Nine units were excluded because they did not meet the inclusion criteria, and five units withdrew after the project commenced, following a decision from their management (three units withdrew after the project pre-test and two units withdrew after the project began). One unit could not provide reassurance that they would not implement a CP if assigned to the control group, and therefore were excluded from the study sample. The remaining 14 units were stratified according to the availability of a stroke unit and to the teaching status of the unit, and the strata were randomized to one of the two arms. A simple randomization procedure was carried out in each stratification sample before the intervention, and patient randomization was carried out using a computer-generated sequence with allocation concealment. Blinding of patients and clinicians was not possible [15]. The CP and UC groups each had seven hospitals assigned to them; both groups contained hospitals with a stroke unit, and both included one teaching hospital. The mean number of beds was 405 in the CP group, and 409 in the UC group.

### Calculation of study sample

A calculation was made to identify the sample size needed to detect a significant difference in the 30-day mortality rate [15]. Because of the mortality of patients with ischemic stroke has been reported to range from 8% to 17%, we expected that a similar difference in our sample would be required to evaluate CP as effective [3,25,26]. We consider this estimate reasonable because in the pilot study that we performed previously to the main trial, we observed an overall in-hospital mortality of 19.76%, which is consistent with the higher levels of mortality reported in the literature [24]. Therefore, as the expected mortality rate of patients with ischemic stroke would be 8% to 17%, within 30 days of the incident we expected that the CP intervention would succeed in limiting mortality to 8%, and would therefore be clinically relevant. Based on this goal, a sample size of 476 patients (238 in each group) was required for the study to have 80% power at the 5% significance level. The sample-size calculation was performed in accordance with standard criteria for a cRCT [16,19,22,27]. The sample size was adjusted using an inflation factor of 1.43 to account for cluster randomization: seven clusters per trial arm, with a cluster size of thirty-four patients and an intra-cluster correlation coefficient (ICC) of 0.018 [15,28-30].

### Participants

The sample included all consecutive patients admitted to the hospitals during the experimental period with a principal diagnosis of acute ischemic stroke (*International Classification Diseases*, Ninth Revision. Clinical Modification (ICD-9CM) code 434.91). To be included, patients had to be at least 18 years of age and admitted within 24 hours of stroke onset. Patients with hemorrhagic strokes (all ICD-9CM codes included in code 431) or transient ischemic attacks (ICD-9CM code 435.9) were excluded. Baseline was verified by comparing the two groups on admission both at the individual level (patient demographics and characteristics) and at the institutional/cluster level (availability of healthcare facilities, technologies, and human resources).

### Randomization and interventions

The project started at each unit with a grand round that outlined the project protocol. To facilitate project implementation, a healthcare worker experienced in CPs was assigned to each unit in the experimental group. The teams included internal-medicine physicians, neurologists, physiatrists, epidemiologists, physiotherapists, occupational therapists, nurses, speech therapists, hospital pharmacists, psychologists, social workers, and support staff. The teams were formed on a voluntary basis; they received 3 days of training in quality improvement and in the development of CPs. To ensure the intervention was standardized, the teams also received a package including evidence-based key interventions and indicators provided by senior investigators of the Evidence-Based Medicine (EBM) unit of the Regional Healthcare Agency of Marche and the University of Eastern Piedmont [31-34]. The teams developed their CPs over a 6-month period (for examples of CPs, see Additional file 1, Additional file 2). All groups analyzed their care processes, reviewed best evidence, defined the appropriate goals of the pathways, and compiled the results into protocols and documentation [14,35-37]. After development, the CPs were analyzed by the EBM unit of the Regional Healthcare Agency of Marche, and were judged consistent with current recommendations for the diagnosis and treatment of stroke. After validation of the CPs, each unit team educated its staff in the use of the CP and monitored its use. This meant that the CPs used in the study were not completely identical because of organizational adaptations at some sites [11]; however, all adhered substantially to existing Italian guidelines on the hospital treatment and rehabilitation of stroke, as described by Gensini *et al. *[33] and Provinciali [38].

### Outcomes and follow-up

The primary outcome measure was 30-day mortality after stroke [15] defined as the proportion of ischemic stroke events that were fatal within 30 days of onset. Stroke fatality was chosen as the main outcome because it is clinically relevant, objectively measured, and reliably coded [8]. The effect of CP intervention was analyzed with respect to 7-day mortality, length of in-hospital stay, hospital re-admission rates, institutionalization rates after hospital discharge, dependency levels, and complication rates along the entire continuum of care [15]. The quality of the care provided to the patients was assessed by monitoring the implementation of evidence-based practice. The implementation of organized care at the cluster level was also evaluated. The list of indicators has been described previously in detail [15].

### Data collection

Local staff prospectively collected the data for both the intervention and the control groups. The staff were not given an incentives for data collection. Staff members were trained in data collection at two educational events. Data were collected using a standardized data-extraction tool that used web technology, and were anonymously entered into a secure database housed at the University of Eastern Piedmont. Mortality data were extracted from the Italian National Register of Mortality, which is based on local registers that are completed and used in each Health Authority at the Healthcare District level. These registers are uploaded in real time when a death occurs, and are matched monthly with the Municipality register for births and deaths. Completion of these registers is compulsory by the doctor certifying the death (deaths both at home or in medical institutions) and there is a national procedure for monitoring the quality and reliability of diagnosis data. In the current study, we did not have any particular problems about reliability of diagnosis of death because we followed up two cohorts of people affected by ischemic stroke, and when a death occurred, we verified the concordance of the cause of death recorded in the register with the pre-existing diagnosis of stroke.; the concordance of the two was 100%.

### Statistical analysis

The study design was based on that described previously by Campbell *et al.*, [17] and on the Consort Statement for cRTCs, and the statistical analysis was performed accordingly [27] by the research team. The Fisher exact and Kruskal-Wallis tests for categorical and continuous variables, respectively, were performed at the cluster level. In addition, differences in the rates of 7-day and 30-day mortality and of return to pre-stroke functioning in daily life between groups and according to each variable under study were evaluated at the individual level using random-effects logistic regression, accounting for the clustering effect [15,39,40]. Variables were included if significant at the 0.10 level (backward approach), with the exception of gender, which was forced to entry. The presence of multicollinearity, interaction, and higher power terms was assessed to check final model validity. Patient outcomes were described according to gender (male); comorbidities (based on their Charlson-Deyo index, patients were categorized as having 0 to 1 or > 1 comorbidities) [41]; medical complications (at least one complication); admission to stroke unit (yes/no); access to organized care (based on organized care index as having 0 to1 or > 1 score) [8]; management by a stroke team (yes/no); use of antithrombotic drugs (antiplatelet or anticoagulant during the stay); and assessment of rehabilitation needs (yes/no). Because the assessment of rehabilitation needs does not have any effect on mortality, it was not included in the model for explaining mortality, but was included in the model for return to functional pre-stroke status. Significance was defined as *P *< 0.05 (two-sided). All analyses were intention-to-treat and were carried out using the software programs Statistica 7.1 (StatSoft, USA) and Stata (version 10; StataCorp LP, College Station, TX, USA).

## Results

### Baseline data

The final sample consisted of 476 patients (238 in each group). As shown in Table 1, patients in the CP group had higher rates of hypertension, peripheral vasculopathy, and dementia rates, and a higher male prevalence, whereas the UC group had higher rates of diabetes and chronic obstructive pulmonary disease. No overall differences were found at the cluster level (Table 2).

**Table 1:** Demographic characteristics^a, b^

Characteristics	Clinical pathway, n = 238	No intervention, n = 238	Between-group difference, OR (95% CI)	*P*-value^b^
Age, years (mean ± SD)	74.5 ± 10.8	74.0 ± 11.7	-	0.61
Male gender	138/238 (58.0) (51.4 to 64.3)	98/238 (41.2) (34.9 to 47.7)	1.97 (1.35 to 2.89)	< 0.001
Previous stroke	50/215 (23.3) (17.8 to 29.5)	57/207 (27.5) (21.6 to 34.2)	0.80 (0.50 to 1.27)	0.32
Current smoking status	29/229 (12.7) (12.4 to 22.5)	25/219 (11.4) (3.9 to 11.0)	1.13 (0.61 to 2.07)	0.77
Hypertension	132/229 (57.6) (51.0 to 64.1)	103/219 (47.0) (40.3 to 53.9)	1.53 (1.04 to 2.27)	0.03
Heart failure	86/229 (37.5) (30.0 to 42.8)	78/219 (35.6) (30.6 to 43.8)	1.09 (0.73 to 1.63)	0.69
Coronary heart disease	21/229 (9.2) (5.8 to 13.7)	29/219 (13.2) (9.1 to 18.5)	0.66 (0.35 to 1.25)	0.18
Peripheral vasculopathy	73/229 (31.9) (25.9 to 38.3)	46/219 (21.0) (15.8 to 27.0)	1.76 (1.12 to 2.76)	< 0.01
Diabetes mellitus	52/229 (22.7) (17.4 to 28.7)	73/219 (31.5) (27.1 to 40.0)	0.59 (0.38 to 0.91)	0.01
Diabetes mellitus (organ pathology)	7/229 (3.1) (1.2 to 6.2)	9/219 (4.1) (1.9 to 7.8)	0.74 (0.24 to 2.20)	0.62
COPD	24/229 (10.5) (6.8 to 15.2)	51/219 (23.3) (17.9 to 29.5)	0.48 (0.28 to 0.83)	< 0.01
Cerebrovascular pathology	60/229 (26.2) (20.6 to 32.4)	82/219 (37.4) (31.0 to 44.2)	0.59 (0.39 to 0.90)	0.01
Pressure ulcer	4/229 (1.8) (0.5 to 4.4)	11/219 (5.0) (2.5 to 8.8)	0.35 (0.09 to 1.22)	0.06
Hemiplegy	16/229 (7.0) (4.0 to 11.1)	22/219 (10.1) (6.4 to 14.8)	0.67 (0.33 to 1.38)	0.31
Nephropathy	10/229 (4.4) (2.1 to 7.9)	13/219 (5.9) (3.2 to 9.9)	0.72 (0.29 to 1.81)	0.52
Dementia	24/229 (10.5) (6.8 to 15.2)	10/219 (6.0) (2.2 to 8.2)	2.45 (1.08 to 5.63)	0.02
Hepatopathy	7/229 (3.1) (1.2 to 6.2)	6/219 (2.7) (1.0 to 5.9)	1.12 (0.33 to 1.82)	1
Cancer				
Solid metastasis	2/229 (0.9) (0.1 to 3.1)	3/219 (1.4) (0.3 to 4.0)	0.63 (0.07 to 4.70)	0.68
Leukemia	2/229 (0.9) (0.1 to 3.1)	3/219 (1.4) (0.3 to 4.0)	0.63 (0.07 to 4.70)	0.68
Lymphoma	1/229 (0.4) (0.0 to 2.4)	0/219 (0.0) (0.0 to 1.7)	1.08 (0.14 to 1.92)	1
Others	11/229 (4.8) (2.4 to 8.4)	19/219 (8.7) (3.8 to 14.7)	0.53 (0.23 to 1.21)	0.10

^a^Because of missing data denominators used to calculate percentages may differ from group totals (clinical pathway versus no intervention).

^b^Values are n/total n (%) (95% CI) unless otherwise indicated.

^c^*P*-values represent comparison of each characteristic between two groups (clinical pathway versus no intervention), and all *P*-values are two-sided.

**Table 2:** Hospital characteristics^a^

Hospital characteristics	Clinical pathway, n = 7^b^	No intervention, n = 7^b^	*P*-value^c^
Setting (University hospital (%)	1 (14.3)	1 (14.3)	1
Healthcare workers dedicated to stroke care, n (mean ± SD)			
Physician	46 (6.6) (0.97)	49 (7.0) (1.29)	0.49
Nurse	80 (11.4) (1.51)	88 (12.6) (1.72)	0.21
Physiotherapist	28 (4.0) (1.29)	24 (3.4) (1.39)	0.44
Auxiliary nurse	11 (1.6) (0.79)	10 (1.4) (0.53)	0.69
Speech therapist	2 (0.3) (0.49)	1 (0.1) (0.38)	0.55
Availability of medical equipment in emergency department, n (%)			
24-hour ED	7 (100)	7 (100)	1
CT brain scan	7 (100)	7 (100)	1
MRI brain scan	5 (71.4)	7 (100)	0.17
Eco-color Doppler	5 (71.4)	7 (100)	0.17
Echocardiography	5 (71.4)	7 (100)	0.17
Transcranic Doppler	4 (57.1)	2 (28.6)	0.35
Availability of continuous monitoring during stay, n (%)			
Electrocardiography	5 (71.4)	6 (85.7)	0.35
Finger-press	5 (71.4)	6 (85.7)	0.35
Saturimeter	5 (71.4)	6 (85.7)	0.35
Pulse oximeter	4 (57.1)	4 (57.1)	1
24-hour investigations			
Hematochemical	5 (71.4)	6 (85.7)	0.35
Neurological	5 (71.4)	4 (57.1)	0.60

COPD, chronic obstructive pulmonary disease; CT, computed tomography; ED, emergency department.

^a^Because of missing data denominators used to calculate percentages may differ from group totals (clinical pathway versus no intervention).

^b^Values are n/total n (%) (95% CI) unless otherwise indicated.

^c^*P*-values represent comparison of each characteristic between two groups (clinical pathway versus no intervention), and all *P*-values are two-sided.

### Outcomes and estimation

Table 3 shows the discharge status of all patients according to the type of treatment received and various outcomes of care. The 7-day and 30-day mortality rates were lower in the CP group, but the difference was not significant, although there was a trend towards significance for the 7-day death rates. There were better results in the CP group for most of the secondary outcomes (rates of return to pre-stroke functioning in daily life, pressure-sore rates, and overall and in-hospital complication rates), except for the average length of stay, which was shorter in the UC group.

**Table 3:** The outcome indicators^a, b^

Variables	Clinical pathway, n = 238	No intervention, n = 238	Between-group difference, OR (95% CI)	*P*-value^c^
In-hospital death rate within 30 days of admission to hospital	18/238 (7.6) (4.5 to 11.7)	25/238 (10.5) (6.9 to 15.1)	0.70 (0.35 to 1.37)	0.34
In-hospital death rate within 7 days of admission to hospital	7/238 (2.9) (1.2 to 6.0)	16/238 (6.7) (3.6 to 10.2)	0.42 (0.15 to 1.11)	0.05
In-hospital death rate within 30 days of stroke attack	18/238 (7.6) (4.5 to 11.7)	25/238 (10.5) (6.9 to 15.1)	0.70 (0.35 to 1.37)	0.34
Post-discharge death rates (1, 3, 6, 12 months after discharge)	3/53 (5.7) (1.2 to 15.7)	0/27 (0.0) (0.0 to 12.8)	1.54 (1.31 to 1.81)	0.55
In-patients length of stay, n (mean ± SD)	229 (11.78 ± 6.6)	219 (10.88 ± 7.9)	-	0.19
Within 9 days of length of stay in hospital patients' rate	94/227 (41.4) (34.9 to 48.1)	114/215 (53.0) (46.1 to 59.8)	1.60 (1.08 to 2.37)	0.02
Pressure-sore incidence rate	4/229 (1.7) (0.5 to 4.4)	12/219 (5.5) (2.9 to 9.4)	0.31 (0.08 to 0.94)	0.04
Overall in-hospital complications rate	53/229 (23.1) (17.8 to 29.2)	67/219 (30.6) (24.6 to 37.2)	0.68 (0.44 to 1.06)	0.09
Overall post-discharge complications rate	12/103 (11.7) (6.2 to 19.5)	0/27 (0.0) (0.0 to 12.8)	1.30 (0.98 to 1.43)	0.07
Institutionalization at discharge	26/92 (28.3) (19.4 to 38.6)	15/64 (23.4) (14.0 to 36.2)	1.29 (0.58 to 2.87)	0.58
In-hospital re-admission rate (within 30 days of discharge)	0/211 (0.0) (NC)	0/194 (0.0) (NC)	NC	NC
Return to pre-stroke functioning in daily life rate (with ADL/case mix adjustment)	97/208 (46.6) (39.1 to 52.9)	22/90 (24.4) (16.0 to 34.6)	2.70 (1.50 to 4.88)	< 0.001
Return to pre-stroke functioning in daily life rate at follow-up at 3 months (with ADL/case mix adjustment)	62/101 (61.4) (51.2 to 70.9)	5/9 (55.5) (21.2 to 86.3)	1.27 (0.27 to 5.92)	0.73

ADL, Activities of daily living; NC, Not calculable

^a^Because of missing data denominators used to calculate percentages may differ from group totals (clinical pathway versus no intervention).

^b^Values are n/total n (%) (95% CI) unless otherwise indicated.

^c^*P*-values represent comparison of each characteristic between two groups (clinical pathway versus no intervention), and all *P*-values are two-sided.

With regard to implementation of evidence-based key interventions into daily practice through the continuum of care, these were much more frequently used for the CP than for the UC groups (Table 4). Similarly, organized care was more frequently used for the CP group (Table 5). The proportion of patients receiving organized care was also significantly higher in the CP group than in the UC group.

**Table 4:** The process indicators^a, b^

Variables	Clinical pathway, n = 238	No intervention, n = 238	Between-group difference, OR (95% CI)	*P*-value^c^
Information, advice, and support from the multidisciplinary team given to the patients (and with their consent, to the caregivers)	204/204 (100) (98.2 to 0.0)	115/133 (86.5) (81.2 to 92.3)	1.16 (1.08 to 1.24)	< 0.001
Use of clinical protocols (see protocol)	221/229 (96.5) (93.2 to 98.5)	83/139 (59.7) (51.1 to 67.9)	18.64 (8.14 to 44.31)	< 0.001
Use of CT/MRI brain scan within 48 hours of admission	223/229 (97.4) (94.4 to 99.0)	209/219 (95.4) (91.8 to 97.8)	1.78 (0.58 to 5.61)	0.31
Aspirin treatment within 48 hours of admission	157/188 (83.5) (77.4 to 88.5)	146/196 (74.5) (67.8 to 80.4)	1.73 (1.02 to 2.75)	0.03
Swallow screen test on day of admission	211/213 (99.1) (96.6 to 99.9)	83/95 (87.4) (79.0 to 93.3)	15.25 (3.14 to 100.96)	< 0.001
Blood pressure assessment	173/197 (87.8) (82.3 to 92.0)	78/191 (40.8) (33.8 to 48.2)	10.44 (6.06 to 18.10)	< 0.001
ECG/ECD within 24 hours of admission	168/181 (92.8) (90.4 to 96.9)	205/218 (94.0) (90.0 to 96.8)	0.82 (0.35 to 1.94)	0.69
Continuous monitoring within 48 hours of admission (see protocol)	85/229 (37.1) (30.8 to 43.7)	21/219 (9.6) (6.0 to 14.3)	5.57 (3.21 to 9.73)	< 0.001
Before discharge total assessment (see protocol)	200/215 (93.0) (89.4 to 96.3)	176/200 (88.0) (85.1 to 93.9)	1.82 (0.88 to 3.77)	0.09
Use of discharge plan (and communication)	73/214 (34.1) (25.9 to 38.3)	41/200 (20.5) (13.8 to 24.5)	2.01 (1.26 to 3.21)	< 0.01
Use of SIGN guideline-based discharge plan	188/207 (90.8) (86.0 to 94.4)	0 (0.0) (0.0 to 3.7)	999.4 (137.0 to 20374.0)	< 0.001
Use of discharge summary and information (information pack)	190/214 (88.8) (77.5 to 87.6)	134/200 (67.0) (54.4 to 67.7)	3.90 (2.26 to 6.67)	< 0.001
Before discharge assessment with FIM scale	201/209 (96.2) (93.4 to 98.5)	76/168 (45.2) (39.0 to 54.6)	30.41 (13.49 to 71.24)	< 0.001
Follow-up assessment at 3 months with FIM scale	82/86 (95.3) (89.9 to 98.9)	9/29 (31.0) (16.1 to 50.0)	45.56 (11.20 to 205.58)	< 0.001

ABC, Airway, Breathing, Circulation; CT, computed tomography; ECD, Eco-color doppler; FIM, Functional independence measure; MRI, magnietic resonance imaging; SIGN, Scottish Intercollegiate Guideline Network.

^a^Because of missing data denominators used to calculate percentages may differ from group totals (clinical pathway versus no intervention).

^b^Values are n/total n (%) (95% CI) unless otherwise indicated.

^c^*P*-values represent comparison of each characteristic between two groups (clinical pathway versus no intervention), and all *P*-values are two-sided.

**Table 5:** The organized care indicators^a, b^

Variables	Clinical pathway, n = 238	No intervention, n = 238	Between-group difference, OR (95% CI)	*P*-value^c^
Admission to stroke unit	132/226 (58.4) (51.7 to 64.9)	32/197 (16.2) (11.4 to 22.2)	7.24 (4.45 to 11.82)	< 0.001
Stay in stroke unit within 24 hours after admission and until the end of in-hospital rehabilitation	94/117 (80.3) (72.0 to 87.1)	4/31 (12.9) (3.6 to 29.8)	27.59 (8.06 to 104.10)	< 0.001
Use of case managers (physiotherapists, occupational therapists, nurses specialized in stroke care)	223/224 (99.6) (97.5 to 100.0)	104/192 (54.2) (46.8 to 61.4)	188.69 (27.93 to 3697.86)	< 0.001
Use of stroke team	220/222 (99.1) (96.8 to 99.9)	82/126 (65.1) (56.1 to 73.4)	59.02 (13.57 to 360.39)	< 0.001
Assessment of rehabilitation needs by a member of the stroke team within 48 hours after admission	217/225 (96.4) (93.1 to 98.5)	126/219 (57.5) (50.7 to 64.2)	20.02 (9.04 to 46.12)	< 0.001
Patients' needs assessment and planning rate for post-discharge services	201/209 (96.2) (92.6 to 98.3)	68/168 (40.5) (33.0 to 48.3)	32.84 (15.08 to 73.81)	< 0.001
Follow-up rate within 3 months after discharge (by specialist/stroke team)	63/64 (98.4) (91.6 to 100.0)	0/27 (0.0) (0.0 to 12.8)	28.00 (4.09 to 191.88)	< 0.001

^a^Because of missing data denominators used to calculate percentages may differ from group totals (clinical pathway versus no intervention).

^b^Values are n/total n (%) (95% CI) unless otherwise indicated.

^c^*P*-values represent comparison of each characteristic between two groups (clinical pathway versus no intervention), and all *P*-values are two-sided.

### Ancillary analyses

The results of the multivariate random-effect logistic models predicting the 7-day and 30-day mortality and the rates of return to pre-stroke functioning in daily life are shown in Table 6. After adjusting for gender, comorbidities, complications, use of stroke unit, organized care, stroke team, and antithrombotic therapy, patients in the CP group had a significantly lower risk of 7-day in-hospital death (OR = 0.10; 95% CI 0.01 to 0.95) and a significantly higher probability of returning to pre-stroke functioning in their daily life (0.42; 0.18 to 0.98) compared with patients in the UC group.

**Table 6:** Multivariate analysis: 7-day and 30-day mortality and pre-stroke status (not return)

	Variables								
	
Outcome	Pathway	Male gender	Comorbidities	Complications	Stroke unit	Organized care	Stroke team	Antithrombotic treatment	Assessment of rehabilitation needs
7-day mortality									
*P *value	**0.04**	0.89	0.85	0.06	0.13	0.65	0.18	0.07	-
Odds ratio	**0.10**	1.14	1.27	4.71	0.24	0.54	0.17	0.21	-
-95% CI	**0.01**	0.19	0.11	0.95	0.04	0.04	0.01	0.04	-
+95% CI	**0.95**	6.75	14.82	23.37	1.52	8.01	2.36	1.16	-
30-day mortality									
*P *value	0.12	0.78	0.15	**0.00**	0.08	0.81	0.12	**0.03**	-
Odds ratio	0.30	0.84	2.79	**7.17**	0.29	0.74	0.15	**0.25**	-
-95% CI	0.06	0.24	0.70	**2.22**	0.07	0.06	0.01	**0.07**	-
+95% CI	1.39	2.92	11.19	**23.15**	1.16	8.54	1.68	**0.85**	-
Pre-stroke status (not return)									
*P *value	**0.04**	0.76	0.81	**0.00**	0.84	0.11	0.08	0.22	0.53
Odds ratio	**0.42**	1.10	1.09	**5.72**	0.93	0.09	0.12	0.62	0.33
-95% CI	**0.18**	0.60	0.54	**2.36**	0.49	0.00	0.01	0.29	0.01
+95% CI	**0.98**	2.00	2.18	**13.87**	1.80	1.73	1.27	1.34	10.85

^b^Values are n/total n (%) (95% CI) unless otherwise indicated.

## Discussion

### Interpretation

The main finding of this study is that care delivered using CPs to patients with stroke was significantly more evidence-based than that delivered to patients with stroke receiving UC, and this seemed to translate to more effective treatment, because implementation of CPs resulted in a significant improvement of the outcomes.

There are several potential (and not mutually exclusive) explanations for these results, ranging from selection bias to improved performance. Selection bias may have occurred both at the individual and cluster levels. At the cluster level, no differences emerged; however, after randomization, some differences were seen between the CP and non-CP groups at the individual level. Therefore, interpretation of the outcomes before randomization could have been biased. Moreover, we could not use the National Institute of Health Stroke Scale (NHISS), because the study started before validation of this instrument in 2009 [42]. Patient assessment before admission was partly performed, therefore the severity of stroke was not properly assessed. Except for mortality measures, we found a significant drop in sample size for certain outcomes in the non-intervention group. Thus, this may limit our findings regarding outcome improvement. However, CP use remained a significant determinant of the improved patient outcomes even after adjusting for potential confounders, including comorbidities at baseline and complications during the hospital stay. Therefore, the influence of selection bias, if any, is likely to be minor [43].

A second possible explanation for our findings is the different use of organized care in the two groups. The use of organized care and the access to the stroke units was were significantly higher in the CP group. Because use of organized care and stroke units are integrated approaches to managing stroke and are strong evidence-based independent predictors of in-hospital mortality, they were used as 'active components' promoted by the implementation of the CP [8,24]. Therefore, the observed improvements in organized care and in use of stroke units and their positive effect on patient outcome were expected as part of the intervention.

### Generalizability

With regard to outcomes, our results show how evidence-based care can be effectively implemented in real-world settings [6,11]. Indeed, the use of evidence-based care was significantly improved in the CP group, which also made better use of the organized care, as mentioned above. These findings were apparent despite the complexity of objective evaluation of stroke, as has been reported by the primary stroke center certification program and the performance measurements of the Joint Commission [44-46]. Our findings were mostly based on short-term outcomes. Long-term and/or more qualitative outcomes, such as patient satisfaction and quality of life, were not evaluated. This was mainly due to the information system, especially in the method of documenting and collecting data (clinical records, paper-based abstraction tools), and could also be a limitation of our findings [12,47].

In the study, we found a significant reduction only in 7-day mortality, whereas 30-day mortality was not significantly affected by the use of CPs. The 7-day mortality depends more on the early treatment of patients, but no specific protocol for factors influencing early arrival was implemented in the CP. Therefore, it is possible that the CP hospitals might have admitted patients earlier, and this would have artificially reduced their short-term in-hospital mortality rate. Although we did not have specific data on the events before admission, it is reasonable to assume that the randomization process would have controlled for this possible bias. However, after risk adjustment, the CP method remained a significant determinant of reduced 7-day mortality. Moreover, at discharge, patients in the CP group had a significantly higher rate of return to pre-stroke functioning compared with the UC group, and the process of care was significantly more consistent with evidence-based care. Based on our findings and those of other authors, we consider it reasonable to believe that the better outcomes observed for the CP group are attributable to an improvement in the quality of care achieved with the use of CPs [11,44,48,49].

A limitation of our findings is that we could not examine the effect of CPs on the use of reperfusion therapy (tissue plasminogen activator; tPA), because in Italy the non-experimental use of this procedure was authorized only after the conclusion of this study. Early treatment based on tPA involves the expertise of several professionals, which can result in poor coordination or inefficiencies in the care process. Indeed, several studies have shown that tPA use in acute ischemic strokes can be enhanced by organized care, routine use of protocols, and multidisciplinary teamwork, which the current study also showed identified as active components of the implemented CP [48,50]. Therefore, our findings suggest that the use of CPs through the implementation of better care might also be helpful for the effective use of tPA, but specific studies are required.

Because CPs are quality improvement initiatives it was not possible to blind the intervention or the assessment of the outcomes. Therefore it could have been that the awareness and attention associated with receiving a new intervention (Hawthorne effect) was responsible, in part, for the improved outcomes associated with the CPs. Given the type of intervention, a cluster randomized design was the most appropriate design to use [19,22,27,40]. However, these studies have some limitations when applied to CPs [51]. Indeed, context level adaptation, which is essential for the pathways to succeed, may be perceived as inappropriate in the trial design. Also, it may be difficult to replicate and maintain the original intervention. To reduce such issues, we implemented pathways that combined local standards with evidence-based indicators to maintain the integrity of the intervention at each site [21,52].

## Conclusions

This study adds evidence in favor of the notion that, compared with UC, CPs can help to provide better, comprehensive, and more specialized care to patients affected by stroke. However, additional studies are needed to further understand the cost-effectiveness of CPs and how to pass the critical point at which adherence to a pathway decreases [53-55].

## Conflict of interests

The authors declare that they have no competing interests.

## Authors' contributions

MP conceived and developed this study, managed the project, and wrote the manuscript. MP had full access to all of the data in the study, and takes responsibility for the integrity of the data and the accuracy of the data analysis. SM helped to design the study in defining the indicator set and contributed to the manuscript. RB assisted in cluster creation and performed the statistical analysis. KV contributed to the evaluation of the findings and to the development of the manuscript. FDS provided input in the project, overviewed all steps of the study design, and undertook the final review of the manuscript. All authors have read and approved the final manuscript.

## Pre-publication history

The pre-publication history for this paper can be accessed here:

http://www.biomedcentral.com/1741-7015/10/71/prepub

## Supplementary Material

Additional file 1 **Flow chart of clinical pathway activities before, during, and after hospitalization of patients**. This file contains a graphical representation of clinical pathway activities to be applied before, during, and after hospitalization, at discharge, and at follow-up, as defined by the working teams. Click here for file

Additional file 2 **List of clinical pathway activities to be applied before, during, and after patient hospitalization as defined by the working teams**. This file contains detailed descriptions of of clinical pathway activities for application before, during, and after the hospitalization, at discharge, and at follow-up, as defined by the working teams. Click here for file
