# Supplementary material for: A cluster randomized trial to assess the effect of clinical pathways for patients with stroke: results of the clinical pathways for effective and appropriate care study
Source: BMC Med. 2012 Jul 10;10:71. doi: 10.1186/1741-7015-10-71 (PMC3403956; doi:10.1186/1741-7015-10-71)
Supplement: Additional file 1 — Flow chart of clinical pathway activities before, during, and after hospitalization of patients. This file contains a graphical representation of clinical pathway activities to be applied before, during, and after hospitalization, at discharge, and at follow-up, as defined by the working teams. [file 1741-7015-10-71-S1.PPT]

## Slide 1
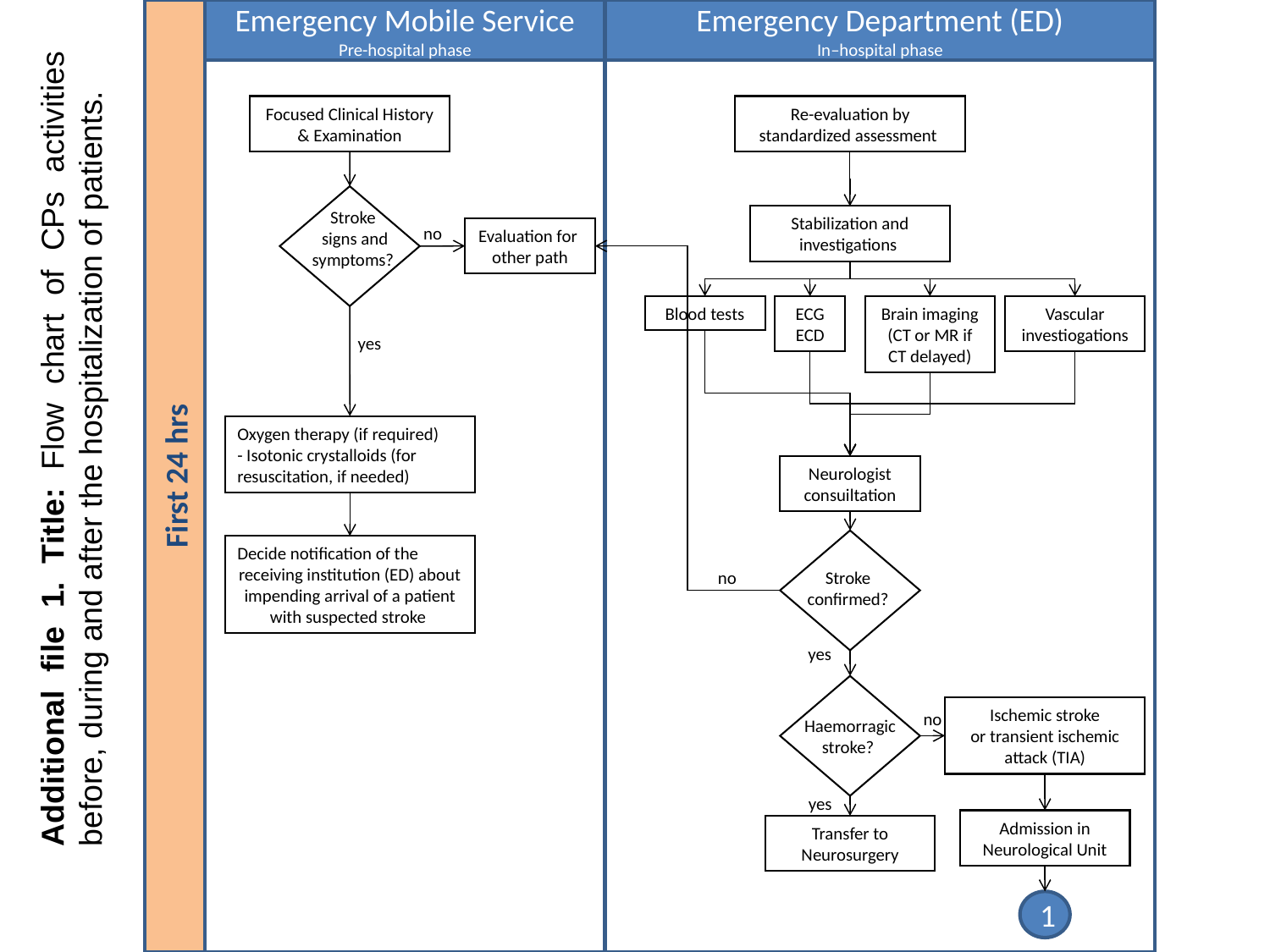

Emergency Mobile Service
Pre-hospital phase
Emergency Department (ED)
In–hospital phase
Focused Clinical History & Examination
Re-evaluation by standardized assessment
Stroke
signs and symptoms?
Stabilization and investigations
no
Evaluation for
other path
Blood tests
ECG
ECD
Brain imaging (CT or MR if CT delayed)
Vascular investiogations
yes
Additional file 1. Title: Flow chart of CPs activities before, during and after the hospitalization of patients.
Oxygen therapy (if required)
- Isotonic crystalloids (for resuscitation, if needed)
First 24 hrs
Neurologist consuiltation
Decide notification of the
receiving institution (ED) about impending arrival of a patient with suspected stroke
no
Stroke
confirmed?
yes
Ischemic stroke
or transient ischemic
attack (TIA)
no
Haemorragic stroke?
yes
Admission in
Neurological Unit
Transfer to
Neurosurgery
1

## Slide 2
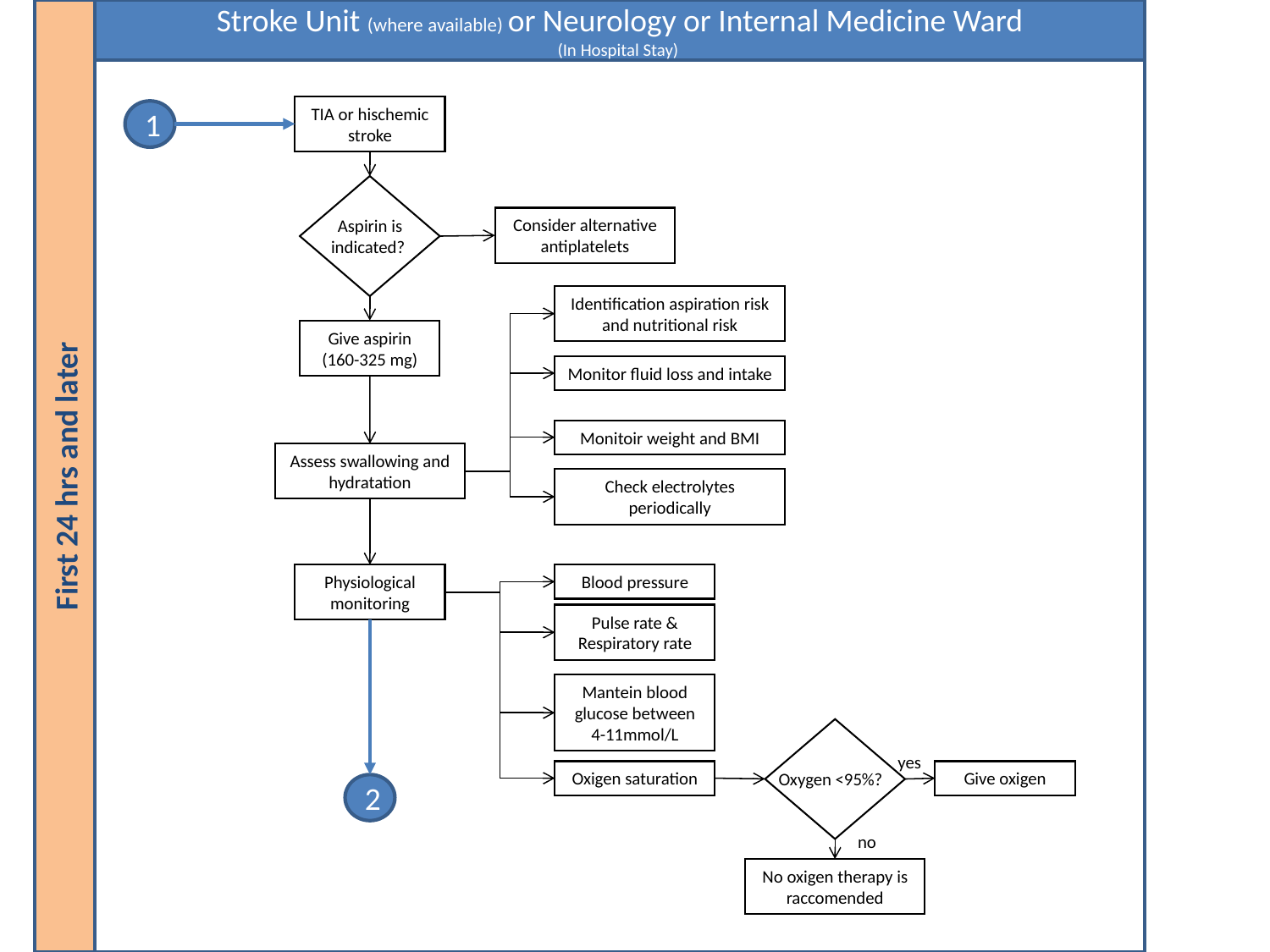

Stroke Unit (where available) or Neurology or Internal Medicine Ward
(In Hospital Stay)
TIA or hischemic stroke
1
Consider alternative antiplatelets
Aspirin is indicated?
Identification aspiration risk
and nutritional risk
Give aspirin (160-325 mg)
Monitor fluid loss and intake
Monitoir weight and BMI
Assess swallowing and hydratation
First 24 hrs and later
Check electrolytes periodically
Physiological monitoring
Blood pressure
Pulse rate &
Respiratory rate
Mantein blood glucose between 4-11mmol/L
yes
Oxigen saturation
Give oxigen
Oxygen <95%?
2
no
No oxigen therapy is raccomended

## Slide 3
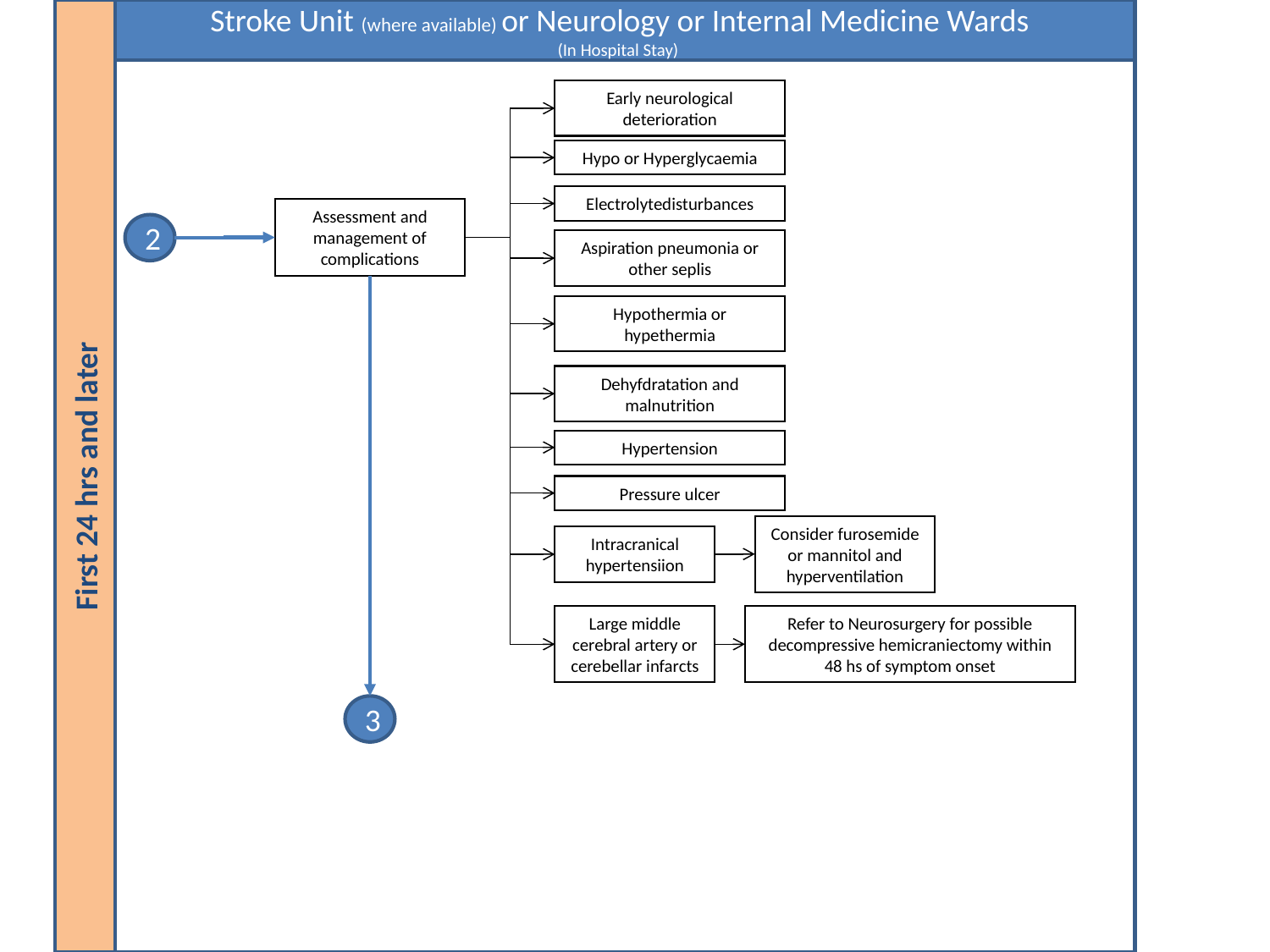

Stroke Unit (where available) or Neurology or Internal Medicine Wards
(In Hospital Stay)
Early neurological deterioration
Hypo or Hyperglycaemia
Electrolytedisturbances
Assessment and management of
complications
2
Aspiration pneumonia or other seplis
Hypothermia or hypethermia
Dehyfdratation and malnutrition
Hypertension
First 24 hrs and later
Pressure ulcer
Consider furosemide or mannitol and hyperventilation
Intracranical hypertensiion
Large middle cerebral artery or cerebellar infarcts
Refer to Neurosurgery for possible decompressive hemicraniectomy within 48 hs of symptom onset
3

## Slide 4
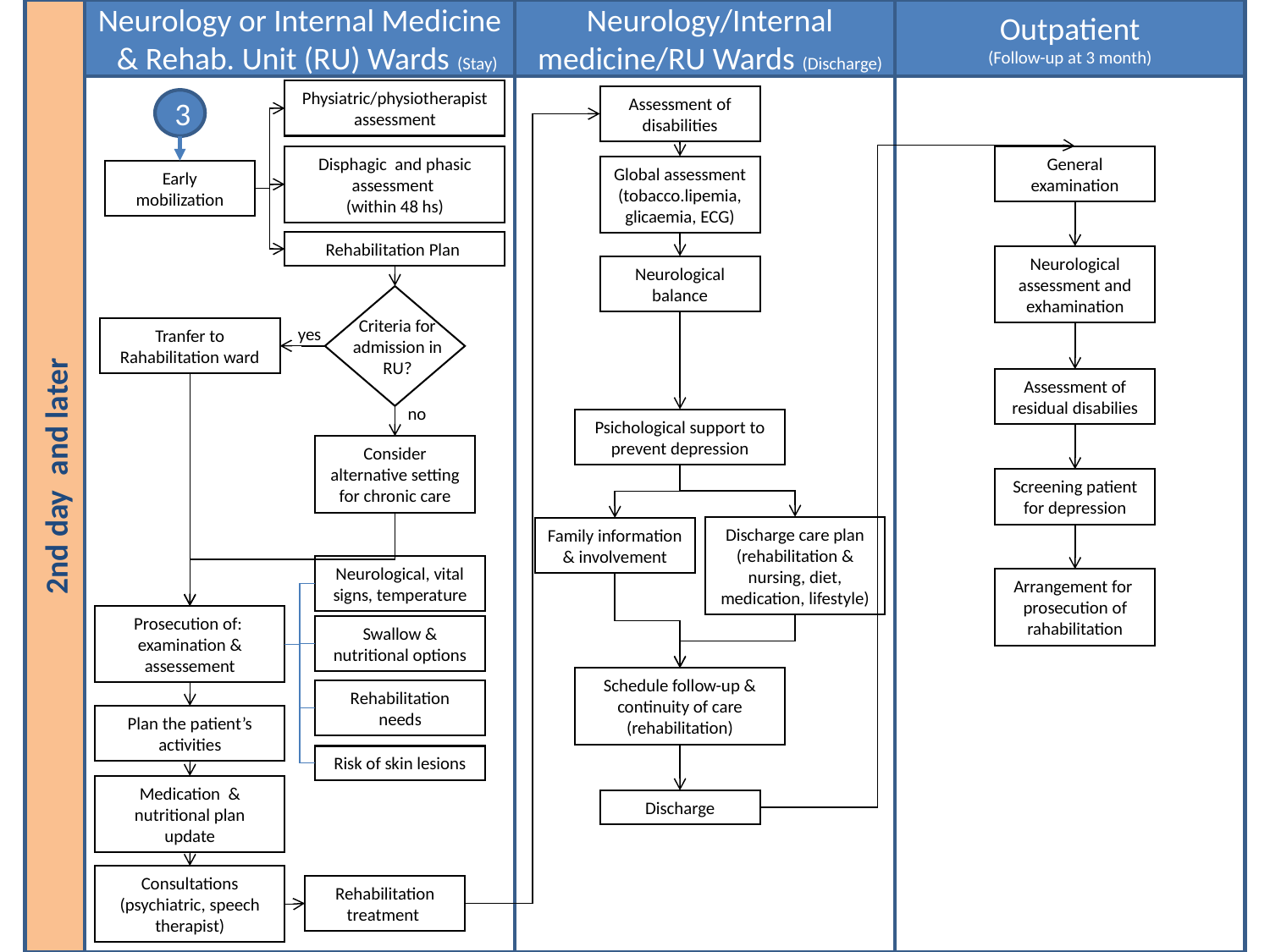

Neurology or Internal Medicine & Rehab. Unit (RU) Wards (Stay)
Neurology/Internal medicine/RU Wards (Discharge)
Outpatient
(Follow-up at 3 month)
Physiatric/physiotherapist
assessment
Assessment of disabilities
3
Disphagic and phasic assessment
(within 48 hs)
General examination
Global assessment (tobacco.lipemia, glicaemia, ECG)
Early mobilization
Rehabilitation Plan
Neurological assessment and exhamination
Neurological balance
Criteria for admission in RU?
yes
Tranfer to Rahabilitation ward
Assessment of residual disabilies
no
Psichological support to prevent depression
Consider alternative setting
for chronic care
2nd day and later
Screening patient for depression
Discharge care plan (rehabilitation & nursing, diet, medication, lifestyle)
Family information & involvement
Neurological, vital signs, temperature
Arrangement for prosecution of rahabilitation
Prosecution of:
examination & assessement
Swallow & nutritional options
Schedule follow-up & continuity of care (rehabilitation)
Rehabilitation needs
Plan the patient’s activities
Risk of skin lesions
Medication & nutritional plan update
Discharge
Consultations (psychiatric, speech therapist)
Rehabilitation treatment
