# Supplementary material for: A cluster randomized trial to assess the effect of clinical pathways for patients with stroke: results of the clinical pathways for effective and appropriate care study
Source: BMC Med. 2012 Jul 10;10:71. doi: 10.1186/1741-7015-10-71 (PMC3403956; doi:10.1186/1741-7015-10-71)
Supplement: Additional file 2 — List of clinical pathway activities to be applied before, during, and after patient hospitalization as defined by the working teams. This file contains detailed descriptions of of clinical pathway activities for application before, during, and after the hospitalization, at discharge, and at follow-up, as defined by the working teams. [file 1741-7015-10-71-S2.DOC]

Additional file 2. Title: List of activities to be applied before, during and after the hospitalization as settled by working teams.

| Healthcare worker | Initial 24 hours | | Day 2 | Day 3 and later | Discharge | Follow-up (at 3 months) |
| --- | --- | --- | --- | --- | --- | --- |
|  | Pre-hospital phase EMS^1^ | In-hospital phase |  |  |  |  |
| Neurologist care  physician and/or, internal care physician | - Focused history and examination  - ABC^2^ evaluation and, vital functions assessment  - Neurologic Recognition of stroke signs and symptoms (GCS^3^, CPSC^4^)  - Cardiac monitoring  - Initiating therapy (if needed)  - Oxygen therapy (if required)  - Isotonic crystalloids (for resuscitation, if needed)  - Decide notification of the  receiving institution (ED^5^) about impending arrival of a patient with suspected stroke | - General physical examination  - Focused history (historical information is the time of symptom onset)  - Stabilization of the ABC  - Assessment of neurological deficits and possible co-morbidities  - Compiling protocols entry and medical record  - Family information  - Requires   - ECG^6^ /ECD^7^ - Blood collection (glucose level, full blood count, urea, electrolytes, creatinine, coagulation profile, erythrocyte sedimentation rate or C-reactive protein, lipid profile, troponine if ECG is abnormal or history of pain) - Brain imaging whit CT scan^8^ (within 1 h if the patient is on anticoagulation therapy, or the patient has a know bleeding tendency, ecc.) - EGA^9^   - Decide:   - diagnosis of ischemic stroke or TIA^10^ (or Haemorragic stroke)   - Prescription therapy  - Required if necessary neurosurgical and cardiologic consultancies  - Choose:   - Drugs - Routes of administration - Schedules of administration   - Swallow assessment  - Assessment and management of complications  - Nutrition management (eg. recommendations on diet)  - Establishes the patient's activity  - Provide safety measures | - Neurological examination and assessment  - General physical examination  - Vital signs assessment  - Compiling medical records  - Assessment of the patient's diagnostic test  - Family information  - Required complementary tests:   - Carotid Doppler ultrasound - Echocardiogram TTE^11^/TEE^12^ - MR^11^ brain scans + MR angiography time-of-flight (TOF)   - CT brain angiography  - Digital subtraction angiography  - TCD^13^  - Blood tests for further diagnostic  - Required:   - Active physiatrist consult - Active speech therapist consult   - Schedule the rehabilitation plan  - Choose:   - Drugs - Routes of administration - Schedules of administration   - Assesses the ability to swallow and nutritional options:   - per os - parenteral - nasogastric tube   - Nutrition management (eg. recommendations on diet)  - Establishes the patient's activity  - Provide safety measures | - Neurological examination and assessment  - General physical examination  - Vital signs assessment  - Compiling medical records  - Assessment of the patient's diagnostic test, requires further tests if necessary (neurological or other)  - Family information  - Required:   - Active physiatrist consult - Active speech therapist consult   - Evaluate the advice based on clinical needs  - Choose:   - Drugs - Routes of administration - Schedules of administration   - Assesses the ability to swallow and nutritional options   - per os - parenteral - nasogastric tube   - Nutrition management (eg. recommendations on diet) | - Neurological balance  - Assess disabilities before discharge with FIM scale^13^  - Use of discharge summary and information (information pack) for patient and family information  - Before discharge total assessment (see parameters below)   - tobacco smoke - lipemia - glycaemia - ECG   - Provide a discharge care plan containing the following:   - Medication regimen - Prescribed diet - Guide patient in lifestyle modification based on identified risk factor   - Use of SIGN^15^ guidelines-based discharge plan (multidisciplinary teamwork discharge plan)  - Medical follow-up schedule | - General physical examination  - Neurological examination and assessment  - Assessment of post-discharge complications  - Medical follow-up schedule |
| Physiatrist |  |  | - Assessment of rehabilitation needs  - Organizes the rehabilitation plan |  | - Final evaluation of disability  - Plan the patient’s activities for continuation rehabilitation | - Assessment of residual disability with FIM scale^14^  - Restore the patient’s activities (if needed) |
| Speech therapist |  |  | - Assessment of aphasia | - Assessment of aphasia | Plan the patient’s activities rehabilitation | - Assessment of residual aphasia  - Restore the patient’s activities (if needed) |
| Epidemiologist |  | - Acquisition of clinical admissions data | - Acquisition of clinical in-hospital data | - Acquisition of clinical in-hospital data | - Acquisition of clinical discharge data | - Acquisition of clinical follow-up data |
| Nurse | - Intravenous access (established)  - Assess blood glucose | - Evaluation:   - degree of patient autonomy - bowel and sphincter functions - risk of skin lesions   - Put the patient to bed  - Ensure the correct position  - Ensure the airway flows  - Detect vital signs  - Monitor (ECG, BP^16^, O2^17^ saturation)  - Monitor temperature  - Monitor neurological signs and, awareness  - Monitor weight and BMI^18^  - Sets the daily worksheet  - Performs blood collection and check ECG periodically  - Active blood glucose profile in case of: hypoglycaemia/hyperglycæmia  - Completion nursing record  - Applies the bladder catheters if they are medically necessary  - Attend the patient for diagnostic tests  - Fluid management  - Administer therapies  - Early and frequent mobilization | - Evaluation:   - degree of patient autonomy - bowel and sphincter functions - risk of skin lesions   - Put the patient to bed  - Ensure the correct position  - Ensure the airway flows  - Detect vital signs  - Monitor temperature  - Monitor (ECG, BP, O2 saturation)  - Monitor neurological signs and, awareness  - Sets the daily worksheet  - Performs diagnostic tests required  - Perform blood glucose profile  - Completion nursing record  - Check the bladder catheter if necessary  - Attend the patient for diagnostic tests  - Monitor hydration (physiological solution)  - Administer the medications  - Runs the diet program  - Monitor position and mobilization periodically | - Evaluation:   - degree of patient autonomy - bowel and sphincter functions - risk of skin lesions   - Put the patient to bed  - Ensure the correct position  - Ensure the airway flows  - Detects vital signs  - Monitor temperature  - Monitor neurological signs and, awareness  - Sets the daily worksheet  - Performs diagnostic tests required  - Perform blood glucose profile  - Completion nursing record  - Check the bladder catheter if necessary  - Attend the patient for diagnostic tests  - Monitoring hydration (physiological solution)  - Administer the medications observing to protocols  - Runs the diet program  - Monitor position and mobilization periodically | - Evaluation:   - degree of patient autonomy - bowel and sphincter functions   - Provide a  discharge nurse care plan  with information about:   - therapy - diet - prevention of skin lesions - compliance to medications - pulmonary toile | - Assist physicians during the assessments  - Assessment of patient and family to prescribed  discharge care plan |
| Head nurse |  | - Coordinate nursing activities | - Coordinate nursing activities | - Coordinate nursing activities | - Coordinate nursing activities |  |
| Physiotherapist and/or Occupational therapist |  |  | - Provide specific care such as:   - correct position - mobilization   - Start the physiotherapy program | - Monitor position and mobilization  - Keep on the physiotherapy program | - Plan the patient’s activities for rehabilitation (as a discharge co-ordinator with multidisciplinary teamwork) | - Restore the patient’s activities (if needed) |
| Psychologist |  |  | - Assist with treatment of adjustment difficulties and other psychological issues | - Assist with treatment of adjustment difficulties and other psychological issues | - Assist with treatment of adjustment difficulties and other psychological issues | - Assessed depression using a validated simple screening test |
| Hospital pharmacist |  |  | - Supports the choice of drugs | - Supports the choice of drugs |  |  |
| Social worker and social family |  |  |  |  | - Help the patient complete everyday functional activities of daily living  - Promotes family involvement in rehabilitation | - Restore the patient’s and family activities |
| Support staff |  | - Managing the hygiene of the patient  - Support other HCW to mobilization and nutrition  - Attend the patient for diagnostic tests | - Managing the hygiene of the patient  - Support other HCW to mobilization and nutrition  - Attend the patient for diagnostic tests | - Managing the hygiene of the patient  - Support other HCW to mobilization and nutrition  - Attend the patient for diagnostic tests |  |  |

**List of abbreviations:**

^1^EMS: Emergency Mobile Service; ^2^ABC: Airway, Breathing, Circulation; ^3^GCS: Glasgow Coma Scale; ^4^ CPSC: Cincinnati Pre-hospital Stroke Scale; ^5^ED: Emergency Department; ^6^ECG: Electrocardiogram;

^7^ECD: Echo color Doppler; ^8^CT scan: Computed tomography; ^9^EGA: Arterial blood gas analysis; ^10^TIA: Transient ischemic attack; ^10^TTE: Trans-thoracic echocardiogram; ^11^TTE: Trans-esophageal echocardiogram;

^12^MR: magnetic resonance; ^13^TCD: Trans-cranial Doppler; ^14^FIM scale: Functional independence measure; ^15^SIGN: Scottish Intercollegiate Guideline Network; ^16^BP: Blood pressure; ^17^O2: Oxygen; ^18^BMI: Body mass Index
